# Organic and inorganic elicitors enhance in vitro regeneration of *Rosa canina*

**DOI:** 10.1186/s43141-021-00166-7

**Published:** 2021-05-03

**Authors:** Leila Samiei, Mahboubeh Davoudi Pahnehkolayi, Ali Tehranifar, Zahra Karimian

**Affiliations:** 1 Research Center for Plant Sciences, Ferdowsi University of Mashhad, Mashhad, 91779948974 Iran; 2 Department of Horticultural Science and Landscape, Faculty of Agriculture, Ferdowsi University of Mashhad, Mashhad, 91779948974 Iran

**Keywords:** Casein hydrolysate, Glutamic acid, In vitro regeneration, Micropropagation, Nodal explant, Silver nitrate

## Abstract

**Background:**

*Rosa canina* is one of the most popular rose species which is widely used as the rootstock for the propagation of rose cultivars. The purpose of the present study is to improve the in vitro propagation efficiency of this valuable plant species using various growth stimulants in a proliferation medium. In this study, in vitro-derived axillary buds of *R. canina* were inoculated in Vander Salm (VS) medium supplemented with varying levels of organic or inorganic elicitors including casein hydrolysate (200, 400, and 600 mg/l), glutamic acid (2, 4, 8, and 12 mg/l), proline (500, 1000, 1500, and 2000 mg/l), and silver nitrate (25, 50, 75, and 100 mg/l), separately. Benzyl amino purine (BAP) as well as naphthalin acetic acid (NAA) were added to all media at a constant rate to promote shoot proliferation.

**Results:**

The results indicated that the supplementation of casein hydrolysate to the VS medium markedly stimulated shoot regeneration by 173% in comparison to control. Shoot proliferation was also positively influenced by glutamic acid at all levels, however, at a lesser extent compared to casein hydrolysate. Silver nitrate at 100 mg/l induced the longest shoots (2.52 ± 0.248 cm) and maximum leaf number (8.90 ± 0.276) among all treatments. Although it did not encourage efficient shoot regeneration, the highest quality shoots with maximum growth vigor were observed in this treatment.

**Conclusion:**

In this study, the promising role of casein hydrolysate in combination with plant growth regulators has been emphasized for the improved efficiency of *R. canina* regeneration protocol. Moreover, the addition of silver nitrate to the culture medium seems vital for enhancing the quality of regenerated shoots. The results of this study could be beneficial either for the further pharmaceutical or biochemical investigations of *R. canina* or commercial purposes for mass propagation of this specimen.

## Background

*Rosa canina*, commonly known as dog rose, is considered as one of the most popular and known species of roses. It grows naturally in many areas of the world, including Asia, the Middle East, Europe, and North America [1]. In Iran, *R. canina* is widely distributed in several regions in north, east, and west of the country. A high genetic diversity of this species has been previously documented in Iran [2]. Fruit of *R. canina* is proved to be rich in vitamin C and antioxidants, making it a valuable source of nutrition with great health benefits for humans [3, 4]. This species is also a popular rootstock for propagation of cut rose cultivars [5]. *R. canina* is typically reproduced through seed in the nature; however, the seed germination rate is reported to be poor in this species. Given the existence of a high level of heterozygosity in rose species, they are generally preferred to be reproduced vegetatively in order to obtain true to type propagules [6]. Today, the tissue culture technique has been broadly employed for clonal propagation of many ornamental plants. This method is a viable tool that allows for the rapid and efficient mass production of uniform and pathogen-free plantlets in a short period of time [7]. So far, many studies have been accomplished considering the generation of appropriate micropropagation protocols for several rose species and cultivars [8–10]. Regarding *R. canina*, a number of studies are available on its in vitro regeneration. These investigations have mainly focused on optimization of nodal explant proliferation using various plant growth regulators including BAP, NAA, gibberellic acid (GA), and 2,4-Dichlorophenoxyacetic acid (2,4-D) or some nutritional elements [11–15]. The available regeneration methods and protocols are required to be optimized in order to efficiently achieve higher quality plantlets. The use of growth additives in combination with plant growth regulators appears to be a promising strategy to enhance the efficacy of available protocols [7]. These additives can either comprise individual amino acids such as proline, glutamine, and arginine or they can be more complex substances including casein hydrolysate and coconut water which are combinations of various amino acids. Previous studies revealed the positive impact of growth additives in the regeneration frequency of several plant species. For instance, the use of 25 to 50 mg/l of casein hydrolysate in combination with 4 mg/l BAP significantly improved shoot regeneration in bananas [16]. Similarly, the addition of 500 mg/l casein hydrolysate into the MS medium having 1 μM BAP induced the maximum shoot number in Neem tree [17]. In another study, 200 mg/l glutamine resulted in a considerable increase in bud proliferation in *Ficus religiosa* [18].

Apart from the organic substances like amino acids and proteins that have a stimulatory impact on in vitro plant regeneration, inorganic chemicals such as silver nitrate have been documented to encourage multiplication frequency in several plant species [19, 20]. This chemical has also played a significant role in several other tissue culture procedures including somatic embryogenesis [21] in vitro flowering [20], genetic transformation [22], and micrografting [23] in plants. Previous documents evidenced that silver nitrate stimulated in vitro multiplication of some rose species and cultivars through its ethylene inhibitory activity [24, 25]; however, no report is available concerning the impact of this chemical on the regeneration efficacy of *R. canina*.

The main objective of the present study is to improve in vitro shoot regeneration of *R. canina* from nodal explants using growth additives including casein hydrolysate, glutamic acid, proline, and silver nitrate as well as to develop an optimized tissue culture protocol for micropropagation of this species.

## Methods

### Plant material and sterilization

Cuttings of *R. canina* were collected from their habitat in Razavi Khorassan province, Iran, in April 2018. The voucher specimen (herbarium code: 38414-FUMH) is preserved at the herbarium of Ferdowsi University of Mashhad (FUMH), Iran. The explants were prepared by first removing the thorns from the stems. Then the stems were excised to approximately 2 cm length explant, each containing one single axillary node. The explants were then rinsed with running tap water for 1 h and subsequently disinfected with ethanol (70%) for 30 s, sodium hypochlorite (2.5%) for 15 min, and mercury (II) chloride (0.1%) for 10 min. Finally, the explants were rinsed thoroughly three times using sterile distilled water. The explants were then cultured in Murashige and Skoog (MS) [26] basal medium containing 3% (w/v) sucrose and 0.7% agar for shoot initiation. pH of all media was adjusted to 5.8 before autoclaving at 121 °C for 20 min at 104 kPa pressure. All the vessels were maintained under ambient culture room at 24 ± 1 °C under white fluorescent tubes (at 37–40 μmol m^−2^ s^−1^) for 16 h photoperiod.

### Shoot induction and multiplication

Newly developed shoots on the primary explants were excised after 6 weeks of culture and cut into 1.5 cm length explants each containing one axillary bud. Explants were then incubated in Van der Salm medium (VS) [27] supplemented separately with various levels of casein hydrolysate (200, 400, and 600 mg/l), glutamic acid (2, 4, 8, and 12 mg/l), proline (500, 1000, 1500, and 2000 mg/l), and AgNO_3_ (25, 50, 75, and 100 mg/l). All of the media contained 1.5 mg/l BAP and 0.1 mg/l NAA to promote shoot proliferation [13].VS medium including 1.5 mg/l BAP and 0.1 mg/l NAA but devoid of any growth additives was considered as control. Subcultures were made at the similar medium with 4 weeks interval. At the end of the experiment (8 weeks), proliferation indices including a number of regenerated shoots and leaves as well as shoot length were recorded.

### Rooting and greenhouse acclimatization

At the end of the proliferation phase, the regenerated shoots (approximately 3 cm length) were separated and transferred to VS medium containing various levels of NAA or IBA (0, 0.3, 0.6, and 0.9 mg/l) for rooting. After 45 days, rooting indices including rooting percentage, root number, and root length were recorded for each treatment. Subsequently, the well-rooted plantlets were transferred to disinfected substrate mixture including cocopeat:perlite (1:1) and maintained in culture room for acclimatization. The acclimatized plants were then transferred to a greenhouse after 10 days and plant survival rates were evaluated after 1 month.

### Experimental design and statistical analysis

All of the experiments were arranged in completely randomized design (CRD). Each treatment involved 5 replicates (four explants for each replicate), and the experiments were repeated two times. The results were expressed as the average of the replications ± standard error (SE). The data was analyzed in SPSS 19 software by one-way analysis of variance (ANOVA) followed by Duncan post hoc test to compare means (*p* ≤ 0.05).

## Results

### Shoot induction and multiplication

**Fig. 1:**
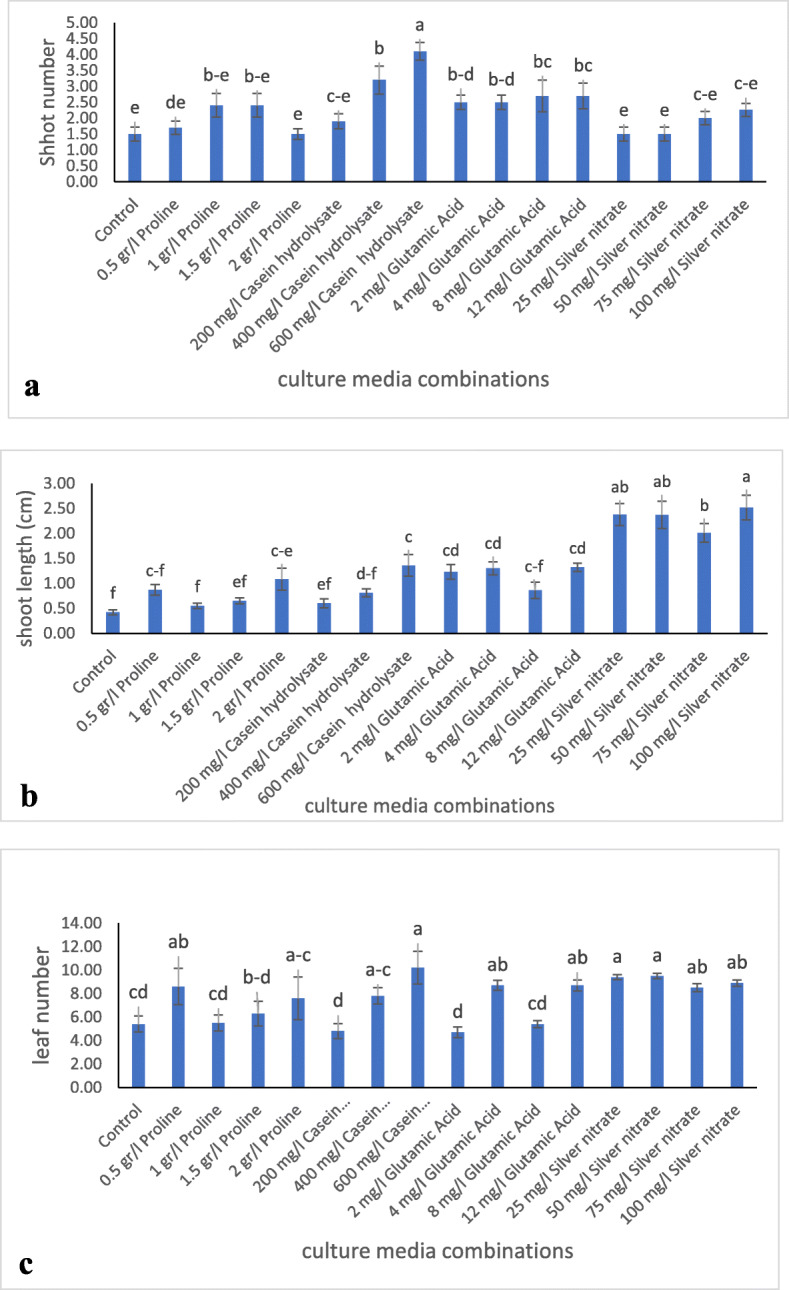
Effect of various growth additives on **a** regenerated shoot number, **b** shoot length, and **c** leaf number of *Rosa canina* nodal explants in vitro condition. Mean values with different letters are significantly different at *p* < 0.05

**Fig. 2:**
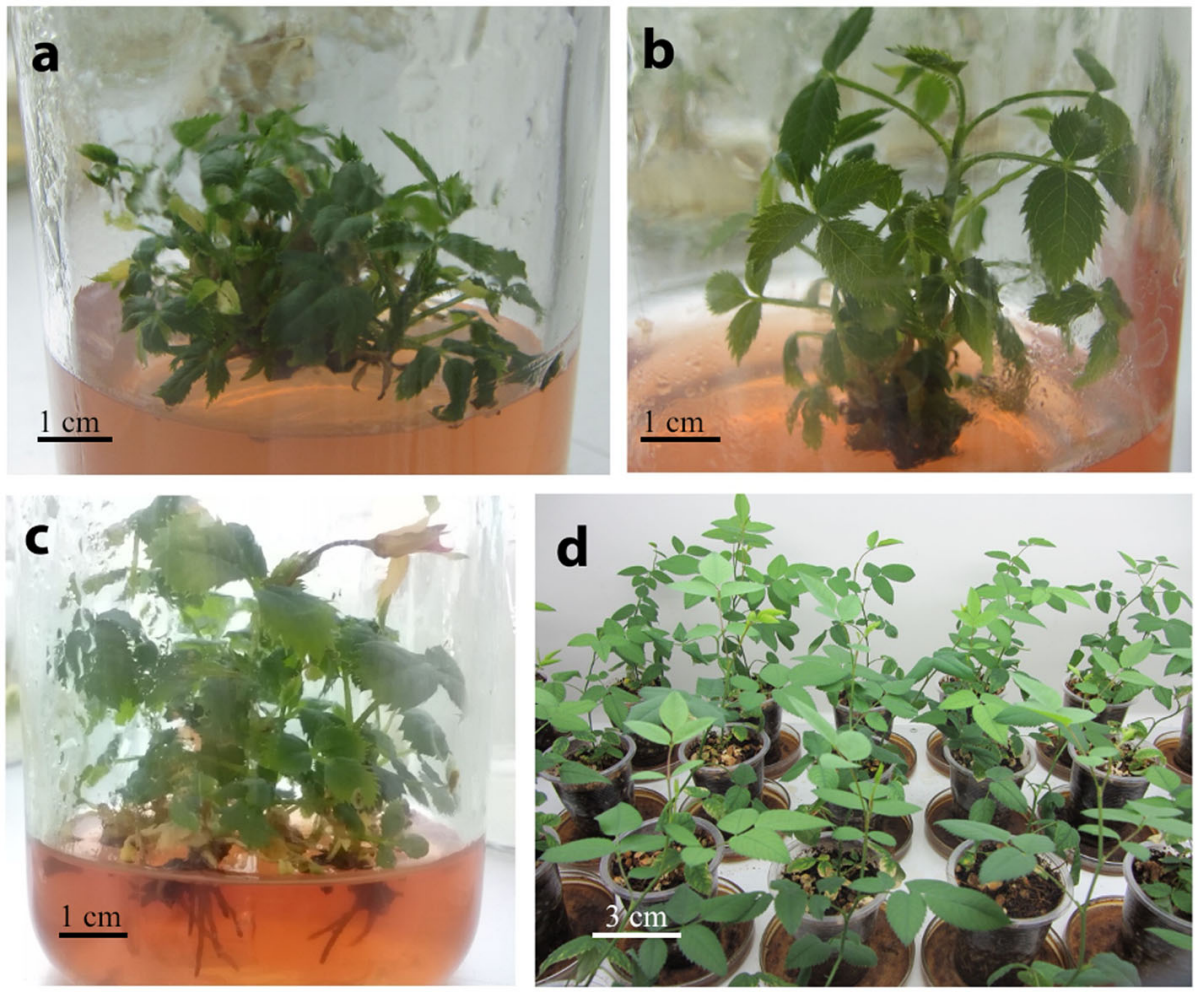
In vitro propagation of *Rosa canina* using nodal explants as affected by various growth additives. **a** Shoot proliferation of nodal explants incubated in VS medium + 600 mg/l casein hydrolysate. **b** Shoot proliferation of nodal explants incubated in VS medium + 50 mg/l silver nitrate. **c** Root induction on VS medium + 0.3 mg/l NAA. **d** Acclimatized plantlets after 1 month in ex vitro condition

The average shoot proliferation rate of *R. canina* was significantly (*P ≤* 0.05) affected by the various growth stimulants after two subcultures (Fig. 1a). The media supplemented with casein hydrolysate or glutamic acid-induced higher regeneration rate in *R. canina* bud explants compared to the rest of treatments. The maximum shoot number of 4.1 ± 0.27 shoots per explant obtained in the medium supplemented with 600 mg/l casein hydrolysate (Fig. 2a). This was 2.5 times higher compared to the shoot number regenerated in the control (1.5 ± 0.22 shoot per explant). All growth indices including shoot number, shoot length, and leaf number with the increase in casein hydrolysate content in the culture medium. Glutamic acid also promoted shoot regeneration in *R. canina* following casein hydrolysate. Twelve milliliters/liter glutamic acid regenerated 80% more shoots as compared to the control. No significant differences were observed in various levels of glutamic acid in shoot proliferation. Nodal explant treated with various concentrations of proline or silver nitrate did not show any significant increase in shoot regeneration compared to the control. The number of regenerated shoots remained minimum in explants incubated in the control as compared to the rest of the treatments.

Silver nitrate in the culture medium allowed for the regeneration of the longest shoot so that at 100 mg/l AgNO_3_, the shoots with an average 2.5 cm length were formed (Fig. 2b). This was approximately 6 times longer than the control (0.42 cm). After the silver nitrate, explants treated with glutamic acid produced the longest shoots compared to control which formed the smallest shoots (Fig. 1b). The maximum number of leaves was obtained in a medium supplemented with 600 mg/l casein hydrolysate (Fig. 1c). This was not significantly different with the leaf numbers produced in media supplemented with either levels of silver nitrate. It should be noted that the most vigorous plant with the highest quality shoots was obtained in the media containing silver nitrate.

### Root induction and greenhouse acclimatization

Following proliferation, regenerated shoots were excised and transferred to rooting media containing varying levels of NAA or IBA. After 1 month, only 3% of the shoots incubated in auxin-free medium (control) were able to regenerate root, whereas, considerably more roots were regenerated in the rest of the treatments possessing various levels of either NAA or IBA (Table 1).

**Table 1:** Effect of various levels of NAA and IBA on in vitro root induction of *Rosa canina*

Culture media combination	Rooting response (%)	Root number	Root length (cm)
Control	3.00 ± 1.50 d	0.30 ± 0.15 d	0.03 ± 0.01 e
0.3 mg/l NAA	43.33 ± 3.07 a	3.9 ± 0.27 a	0.83 ± 0.02 c
0.6 mg/l NAA	23.33 ± 1.11 bc	2.10 ± 0.10 bc	0.93 ± 0.01 bc
0.9 mg/l NAA	15.55 ± 1.81 c	1.40 ± 0.16 c	0.57 ± 0.04 d
0.3 mg/l IBA	28.28 ± 5.05 b	2.54 ± 0.45 b	1.36 ± 0.17 a
0.6 mg/l IBA	15.55 ± 1.81 c	1.40 ± 0.16 c	0.85 ± 0.02 c
0.9 mg/l IBA	17.78 ± 1.81 c	1.60 ± 0.16 c	1.13 ± 0.03 b

Note: Mean values by the different letters are significantly different at *p* < 0.05

Lower concentrations of NAA and IBA proved to be better in rhyzogenesis of regenerated shoots of *R. canina* compared to higher concentrations. Shoots treated with 0.3 mg/l NAA displayed the maximum rooting percentage and root number, 43.32 ± 3.07% and 3.9 ± 0.27, respectively (Fig. 2c), while the shoots subjected to 0.3 mg/l IBA mediated the longest roots (1.36 ± 0.17 cm) in *R. canina* shoots. Rooted plantlets were successfully acclimatized and established in the greenhouse condition with more than 95% survival rate (Fig. 2d).

## Discussion

In the present study, the effects of growth stimulants including casein hydrolysate, proline, glutamic acid, and silver nitrate on shoot proliferation of *R. canina* were investigated. These substances are generally utilized as potential additives besides plant growth regulators during various stages of in vitro plant propagation for the purpose of improving the plant quality and regeneration rate and thereby enhance the efficacy of this process [28]. In this study, plants treated with casein hydrolysate displayed the maximum regeneration rate in *R. canina*. Casein hydrolysate is a mixture of organic substances including low molecular weight proteins, amino acids, vitamins, and growth-stimulating agents that enhance plant growth through facilitating nitrogen availability for the plants [7]. It has been shown that plant cells have a higher ability to metabolize and transfer nitrogen from organic rather than inorganic sources [29]. Casein hydrolysate has been reported to be effective in various plant developmental processes including somatic embryogenesis [30, 31], seed germination and seedling growth [32], and callus proliferation [33]. Additionally, the positive role of casein hydrolysate, as a reduced nitrogen form *in vitro* shoot regeneration of many plant species has been documented previously [34–36].

A range of negative symptoms including shoot tip necrosis and vitrification [37], as well as growth retardation [34, 36], has been reported in some plant species at the high levels of casein hydrolysate (200–500 mg/l). In contrast, none of these responses was observed in our experiment when we used the highest level of casein hydrolysate (600 mg/l). The plants maintained their quality in the maximum level of this substance.

Glutamic acid at 12 mg/l enhanced shoot regeneration and leaf number compared to the control. Glutamic acid is a type of amino acid which is involved in cell function maintenance in plants [38]. It has been effective in induction, maturation, and germination of somatic embryos in a number of plant species [39, 40]. Glutamic acid is the precursor of L-glutamine, which has been frequently reported to have a stimulatory impact on in vitro plant growth, shoot proliferation [38, 39], and somatic embryogenesis [31]. L-glutamine and glutamic acid are directly involved in NH_4_ assimilation in plants. The direct incorporation of these amino acids into culture medium enhances the usage of ammonium and nitrate in plant and thereby facilitate their conversion into amino acids [7]. In the present study, all concentrations of glutamic acid showed a better response in terms of shoot regeneration and length in *R. canina* compared to the controls. These results are consistent with earlier reports confirming the beneficial role of these amino acids on in vitro shoot growth and proliferation on various plant species [41–43].

Proline had no visible impact on the shoot proliferation of nodal explant of *R. canina*. This amino acid was previously reported to be effective in somatic embryogenesis in certain plant species [44, 45]. Although proline at the maximum concentration (2 g/l) improved shoot length and leaf number, it failed to stimulate shoot regeneration in the nodal explant of *R. canina* and thus it is not suggested to be used for direct in vitro regeneration of this plant.

Silver nitrate, although not promoted multiple shoot regeneration in *R. canina*, produced plants with the highest leaf number and shoot length. Moreover, the plants were of the most quality in this treatment. Silver nitrate has been shown to mitigate ethylene biosynthesis while it promotes internal polyamines which consequently enhance cell division and proliferation in plants [46]. Moreover, this substance declines the vitrification rate in plants through encouraging water loss and increasing antioxidant activity [47]. These factors may allow for the regeneration of higher quality plantlets in *R. canina* in the presence of silver nitrate. So far, silver nitrate has been reported to favor plant regeneration from leaf explant of *Rosa* x *hybrida* [24], shoot proliferation in *R. multiflora* [48], and *Rosa* x *hybrida* [49] as well as alleviating leaf chlorosis and necrosis in *R. clinophylla* [50]. In the present study, silver nitrate markedly enhanced plant quality and shoot length. These results are in line with the finding of Ozden et al. (2005) [51] who reported the longer shoots of pistachio in response to addition of silver nitrate in culture medium. In contrasts to our findings, silver nitrate proved to significantly increase in vitro shoot regeneration in certain woody plant species like Joojooba [47] and Prunus [52].

Previous studies indicated that the medium devoid of plant growth regulators was appropriate for in vitro rooting of *Rosa* x *hybrida* cultivars [24]; however, our study indicated that a low concentration of auxin is essential for root regeneration of *R. canina* as poor rooting was observed in auxin-free medium. Ambros et al. (2016) [14] indicated that 1 mg/l IAA was effective in root induction of *R. canina*. Contrastingly, our findings showed that NAA at 0.3 mg/l was the best for the highest root regeneration of this species. IBA or NAA at various concentrations had been effective in in vitro rooting of other rose species so far [50, 53]. These synthetic auxins have been reported to act as synergistic agents with IAA as natural auxin in plants [14].

## Conclusion

This study is the first report where an efficient method for micropropagation of *Rosa canina* was described using organic as well as inorganic growth additives in combination with plant growth regulators. Inclusion of casein hydrolysate in proliferation media increasingly enhanced shoot regeneration in this species. In addition, our study proved that silver nitrate plays a significant role in improving the quality of regenerated shoot in vitro condition. The developed regeneration system in this study could contribute to the commercial production of this economically and medicinally valuable species irrespective of seasonal restriction. Moreover, this system would be of benefit to in vitro breeding purposes of *R. canina* to provide sufficient plant materials for further pharmaceutical, physiological, and biochemical investigations.

## Data Availability

The datasets used during the current study are available from the corresponding author on request.

## Abbreviations

BAP: Benzyl amino purine
CRD: Completely randomized design
GA: Gibberellic acid
MS: Murashige and Skoog
NAA: Naphthalin acetic acid
SE: Standard error
VS: Van der Salm

## References

[1] Nilsson O, Nilsson O, Davis PH (1997). Rosa. Flora of Turkey and the East Aegean Islands.

[2] Samiei L, Naderi R, Bushehri AA, Mozaffarian V, Esselink D, Kazempour S, Smulders MJM (2010). Genetic diversity and genetic similarities between Iranian rose species. J Hortic Sci Biotechnol.

[3] Chrubasik C, Roufogalis BD, Müller-Ladner U, Chrubasik S (2008). A systematic review on the *Rosa canina* effect and efficacy profiles. Phytother Res.

[4] Gruenwald J, Uebelhack R, Moré MI (2019). *Rosa canina*-rose hip pharmacological ingredients and molecular mechanics counteracting osteoarthritis–a systematic review. Phytomed.

[5] Leus L, Van Laere K, De Riek J, Van Huylenbroeck J, Van Huylenbroeck J (2018). Rose. Ornamental crops.

[6] Khosh-Khui M, Teixeira da Silva J (2006). *In vitro* culture of the *Rosa* species. Floric Ornam Plant Biotechnol.

[7] George EF, Hall MA, De Klerk G-J, George EF, Hall MA, De Klerk GJ (2008). The components of plant tissue culture media II: organic additions, osmotic and pH effects, and support systems. Plant propagation by tissue culture.

[8] Malik M, Warchoł M, Kwaśniewska E, Pawłowska B (2017). Biochemical and morphometric analysis of *Rosa tomentosa* and *Rosa rubiginosa* during application of liquid culture systems for *in vitro* shoot production. J Hortic Sci Biotechnol.

[9] Pourhosseini L, Kermani MJ, Habashi AA, Khalighi A (2013). Efficiency of direct and indirect shoot organogenesis in different genotypes of *Rosa hybrida*. Plant Cell Tissue Organ Cult.

[10] Wojtania A, Matysiak B (2018). *In vitro* propagation of *Rosa ‘Konstancin’*(*R. rugosa*× *R. beggeriana*), a plant with high nutritional and pro-health value. Folia Hortic.

[11] Shirdel M, Motallebi-Azar A, Mahna N (2010). *In vitro* micropropagation of dog rose (*Rosa canina* L.). Acta Hortic.

[12] Vântu S (2011). *In vitro* multiplication of *Rosa canina* L. Analele ştiinţifice ale Universităţii Al. I. Cuza Iaşi Tomul LVII Fasc.

[13] Davoudi Pahnekolayi M, Samiei L, Tehranifar A, Shoor M (2015). The effect of medium and plant growth regulators on micropropagation of dog rose (*Rosa canina* L.). J Plant Mol Breed.

[14] Ambros EV, Vasilyeva OY, Novikova T (2016). Effects of *in vitro* propagation on ontogeny of *Rosa canina* L. micropropagated plants as a promising rootstock for ornamental roses. Plant Cell Biotechnol Mol Biol.

[15] Moradian M, Bagheri A (2019). Effect of media composition and plant growth regulators on *in vitro* regeneration of *Rosa canin*a and *Rosa beggeriana*. J Plant Res.

[16] Silue O, Kouassi KM, Koffi KE, Kouakou KEP, Ake S (2017). Effect of adenine sulphate, casein hydrolysate and spermidine on *in vitro* shoot multiplication of two banana varieties (FHIA-21 and PITA-3). Afr J Biotechnol.

[17] Priyanka S, Mithilesh S, Rakhi C (2009). Effect of casein hydrolysate and major inorganic salts on axillary-bud proliferation from nodal explants of a mature neem tree, *Azadirachta indica* Juss. Res J Biotechnol.

[18] Siwach P, Gill AR (2011). Enhanced shoot multiplication in *Ficus religiosa* L. in the presence of adenine sulphate, glutamine and phloroglucinol. Physiol Mol Biol Plants.

[19] Jaberi M, Azadi P, Gharehyazi B, Khosrowchahli M, Sharafi A, Aboofazeli N, Bagheri H (2018). Silver nitrate and adenine sulphate induced high regeneration frequency in the recalcitrant plant *Cosmos bipinnatus* using cotyledon explants. J Hortic Sci Biotechnol.

[20] Panigrahi J, Dholu P, Shah TJ, Gantait S (2018). Silver nitrate-induced *in vitro* shoot multiplication and precocious flowering in *Catharanthus roseus* (L.) G. Don, a rich source of terpenoid indole alkaloids. Plant Cell Tissue Organ Cult.

[21] Al-Khayri JM, Jain SM, Gupta P (2018). Somatic embryogenesis of date palm (*Phoenix dactylifera* L.) from shoot tip explants. Step wise protocols for somatic embryogenesis of important Woody plants, vol 1.

[22] Sgamma T, Thomas B, Muleo R (2015). Ethylene inhibitor silver nitrate enhances regeneration and genetic transformation of *Prunus avium* (L.) cv Stella. Plant Cell Tissue Organ Cult.

[23] Davoudi Pahnekolayi M, Tehranifar A, Samiei L, Shoor M (2019). Optimizing culture medium ingredients and micrografting devices can promote *in vitro* micrografting of cut roses on different rootstocks. Plant Cell Tissue Organ Cult.

[24] Ibrahim R, Debergh PC (2001). Factors controlling high efficiency adventitious bud formation and plant regeneration from *in vitro* leaf explants of roses (*Rosa hybrida* L.). Sci Hortic.

[25] Pati PK, Sharma M, Sood A, Ahuja PS (2004). Direct shoot regeneration from leaf explants of *Rosa damascena* mill in vitro. Cel Dev Biol Plant.

[26] Murashige T, Skoog F (1962). A revised medium for rapid growth and bio assays with tobacco tissue cultures. Physiol Plant.

[27] Van der Salm TP, Van der Toorn CJ, Ten Cate CHH, Dubois LA, De Vries DP, Dons HJ (1994). Importance of the iron chelate formula for micropropagation of *Rosa hybrida* L.‘Moneyway’. Plant Cell Tissue Organ Cult.

[28] Sridhar TM, Aswath CR (2014). Influence of additives on enhanced *in vitro* shoot multiplication of *Stevia Rebaudiana* (Bert.)—an important anti diabetic medicinal plant. Am J Plant Sci.

[29] Persson J, Gardeström P, Näsholm T (2006). Uptake, metabolism and distribution of organic and inorganic nitrogen sources by *Pinus sylvestris*. J Exp Bot.

[30] Baskaran P, Moyo M, Van Staden J (2014). *In vitro* plant regeneration, phenolic compound production and pharmacological activities of *Coleonema pulchellum*. South Africa J Botany.

[31] Lall S, Mandegaran Z, Roberts A (2006). Shoot multiplication and adventitious regeneration in *Sorbus aucuparia*. Plant Cell Tissue Organ Cult.

[32] Li Y-Y, Chan C, Stahl C, Yeung EC, Lee YI, Yeung EC (2018). Recent advances in orchid seed germination and micropropagation. Orchid propagation: from laboratories to greenhouses—methods and protocols.

[33] Salehi M, Moieni A, Safaie N (2017). A novel medium for enhancing callus growth of hazel (*Corylus avellana* L.). Sci Rep.

[34] Baskaran P, Ncube B, Van Staden J (2012). *In vitro* propagation and secondary product production by *Merwilla plumbea* (Lindl.) Speta. Plant Growth Regul.

[35] Jain N, Babbar SB (2003). Regeneration of juvenile plants of black plum, Syzygium cuminii Skeels, from nodal explant of mature trees. Plant Cell Tissue Organ Cult.

[36] Walia N, Kaur A, Babbar SB (2007). An efficient, *in vitro* cyclic production of shoots from adult trees of *Crataeva nurvala* Buch. Ham. Plant Cell Rep.

[37] Daniel MA, David RHA, Caesar SA, Ramakrishnan M, Duraipandiyan V, Ignacimuthu S, Al-Dhabi N (2018). Effect of l-glutamine and casein hydrolysate in the development of somatic embryos from cotyledonary leaf explants in okra (*Abelmoschus esculentus* L. monech). South Africa J Bot.

[38] Newsholme P, Lima M, Procópio J, Pithon-Curi T, Bazotte R, Curi R (2003). Glutamine and glutamate as vital metabolites. Braz J Med Biol Res.

[39] Mazri MA, Meziani R, El Fadile J, Ezzinbi A-E (2016). Optimization of medium composition for *in vitro* shoot proliferation and growth of date palm cv. Mejhoul. 3 Biotech.

[40] Sun Y-L, Hong S-K (2010). Effects of plant growth regulators and L-glutamic acid on shoot organogenesis in the halophyte *Leymus chinensis* (Trin.). Plant Cell Tissue Organ Cult.

[41] Ceasar SA, Ignacimuthu S (2010). Effects of cytokinins, carbohydrates and amino acids on induction and maturation of somatic embryos in kodo millet (*Paspalum scorbiculatum* Linn.). Plant Cell Tissue Organ Cult.

[42] Efzueni Rozali S, Rashid KA, Mat Taha R (2014). Micropropagation of an exotic ornamental plant, Calathea crotalifera, for production of high quality plantlets. Sci World J.

[43] Muthukumar M, Kumar TS, Rao MV (2016). Organogenesis and evaluation of genetic homogeneity through SCoT and ISSR markers in *Helicteres isora* L., a medicinally important tree. South Africa J Bot.

[44] Amini M, Deljou A, Nabiabad HS (2016). Improvement of *in vitro* embryo maturation, plantlet regeneration and transformation efficiency from alfalfa (*Medicago sativa* L.) somatic embryos using Cuscuta campestris extract. Physiol Mol Biol Plants.

[45] Bahmankar M, Mortazavian MM, Tohidfar M, Sadat Noori A, Izadi Darbandi A, Corrado G, Rao R (2017). Chemical compositions, somatic embryogenesis, and somaclonal variation in cumin. Biomed Res Int.

[46] Bais HP, Sudha GS, Ravishankar GA (2000). Putrescine and silver nitrate influences shoot multiplication, *in vitro* flowering and endogenous titers of polyamines in *Cichorium intybus* L. cv. Lucknow local. J Plant Growth Regul.

[47] Gao H, Xu P, Li J, Ji H, An L, Xia X (2017). AgNO_3_ prevents the occurrence of hyperhydricity in *Dianthus chinensis* L. by enhancing water loss and antioxidant capacity. In Vitro Cell Dev Biol Plant.

[48] Rosu A, Skirvin R, Bein A, Norton M, Kushad M, Otterbacher A (1995). The development of putative adventitious shoots from a chimeral thornless rose (*Rosa multiflora* Thunb. Ex J. Murr.) *in vitro*. J Hortic Sci.

[49] Park JS, Naing AH, Kim CK (2016). Effects of ethylene on shoot initiation, leaf yellowing, and shoot tip necrosis in roses. Plant Cell Tissue Organ Cult.

[50] Misra P, Chakrabarty D (2009). Clonal propagation of *Rosa clinophylla* Thory. Through axillary bud culture. Sci Hortic.

[51] Ozden-Tokatli Y, Ozudogru E, Akcin A (2005). *In vitro* response of pistachio nodal explants to silver nitrate. Sci Hortic.

[52] Escalettes V, Dosba F (1993). *In vitro* adventitious shoot regeneration from leaves of *Prunus* spp. Plant Sci.

[53] Akhtar G, Jaskani MJ, Sajjad Y, Akram A (2016). Effect of antioxidants, amino acids and plant growth regulators on *in vitro* propagation of *Rosa centifolia*. Iran J Biotechnol.

